# Oncogenic magnesium transporter 1 upregulates programmed death-1-ligand 1 expression and contributes to growth and radioresistance of glioma cells through the ERK/MAPK signaling pathway

**DOI:** 10.1080/21655979.2022.2037214

**Published:** 2022-04-13

**Authors:** Yuanyuan Wu, Hongbing Wang, Dongdong Wei

**Affiliations:** Department of Tumor Radiotherapy, Cangzhou Central Hospital, Cangzhou, P. R. China

**Keywords:** Glioma, magnesium transporter 1, ERK signaling pathway, MAPK, programmed death-1-ligand 1, radioresistance, tumor growth

## Abstract

Radiotherapy has been established as a major therapeutic modality for glioma, whereas new therapeutic targets are needed to prevent tumor recurrence. This study intends to explore the regulatory role of magnesium transporter 1 (MAGT1) in radiotherapy resistance of glioma through modulating ERK and programmed death-1-ligand 1 (PD-L1). Our bioinformatics analysis identified differentially expressed MAGT1 in glioma, expression of which was subsequently determined in cohort data of TCGA database and microarray dataset as well as glioma cell lines. Artificial modulation of MAGT1, ERK, and PD-L1 expression was performed to examine their effects on glioma cell proliferation and radioresistance, as reflected by MTT and colony formation assays under irradiation. Mouse glioma cells with manipulated MAGT1 and ERK inhibitors were further injected into mice to assess the *in vivo* tumor formation ability of glioma cells. It was noted that MAGT1 expression was highly expressed in glioma tissues of TCGA data and microarray dataset, which was then validated in glioma cell lines. Ectopic expression of MAGT1 was revealed to promote the proliferation and radioresistance of glioma cells, which was attributed to the MAGT1-mediated activation of the ERK/MAPK signaling pathway. It was illuminated that MAGT1 stimulated PD-L1 expression through the ERK/MAPK pathway and thus facilitated glioma cell growth. Additionally, MAGT1 overexpression accelerated the *in vivo* tumor formation of glioma cells, while the ERK inhibitor negated its effect. In conclusion, MAGT1 enhances the growth and radioresistance of glioma cells through the ERK/MAPK signaling pathway-mediated upregulation of PD-L1 expression.

## Graphical Abstract

**Figure:**
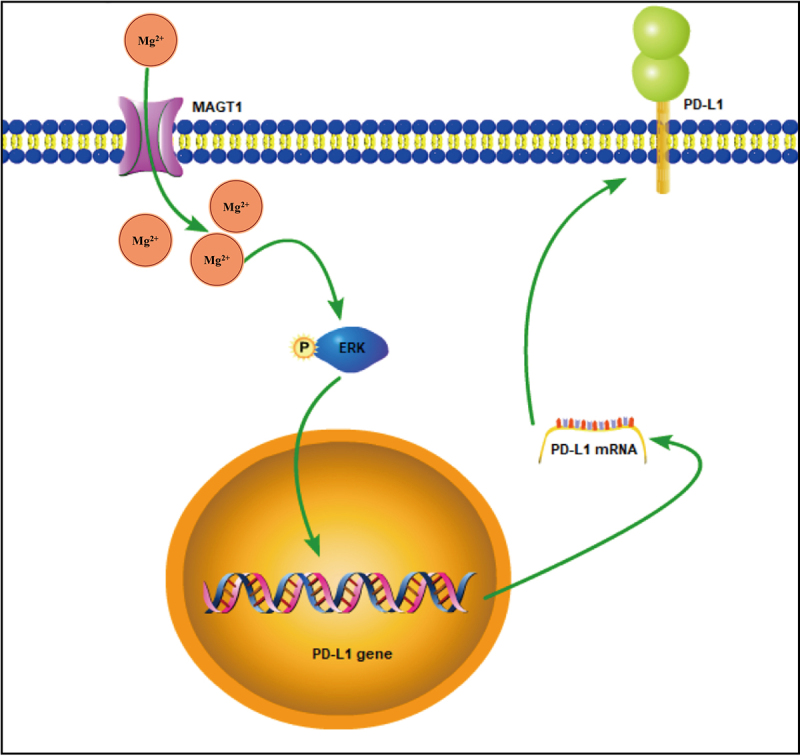

## Introduction

Glioma as the most prevalent central nervous system tumor accounts for almost 40% of the morbidity and mortality caused by brain tumors [1]. Gliomas are historically classified based on the degree of anaplasia they exhibit, known as ‘grade’, and those belong to or greater than grade II are recognized as malignant gliomas due to their locally invasive behavior [2]. For high-grade gliomas, radiotherapy shows rather limited efficacy [3]. Radioresistance is a well-recognized property of glioma cells and has been associated with the poor clinical outcomes of gliomas [4]. In this regard, efforts should be made to explore the molecular mechanisms involved in the radioresistance of glioma cells.

Magnesium transporter 1 (MAGT1) is highly selective for the transportation of Mg [5]. Intriguingly, the overexpression of MAGT1 has been implicated in the occurrence and progression of glioma [6]. Furthermore, a previous report has pointed out that MAGT1 could activate the extracellular signal-regulated kinase (ERK) signaling pathway [7], which may consequently trigger the proliferation and invasion of glioma cells [8,9]. Notably, ERK activation has also been involved in the resistance of glioma cells to radiotherapy [10].

Following the hypothetic MAGT1/ERK regulatory axis, we then noted that the ERK pathway has been established as a mediator of programmed death-1-ligand-1 (PD-L1) in various malignancies such as non-small cell lung cancer and renal cancer [11,12]. Further, intracellular PD-L1 has been reported to confer glioblastoma multiforme malignancy *via* activation of the Ras/ERK/EMT axis [13]. PD-L1 represents an immune checkpoint able to attenuate the potential of T lymphocytes to recognize antigens [14]. Emerging evidence has suggested the prognostic and therapeutic promise of PD-L1 in glioma [15–17]. PD-L1 targeting with immunovirotherapy has been demonstrated to trigger a potent anti-tumor immune response in glioma models [18].

Given the aforementioned evidence, we proposed a hypothesis that MAGT1 in glioma may regulate the ERK/MAPK signaling pathway and the expression of PD-L1, by which mechanism radioresistance of glioma cells may be affected. Investigations were performed *in vitro* and *in vivo* in the present study to testify the hypothesis with the purpose of deepening the understanding of glioma cell radioresistance.

## Materials and methods

### Ethics statement

Animal experiments were approved by the Animal Care and Use Committee of Cangzhou Central Hospital and performed in accordance with *Guide for the Care and Use of Laboratory Animals* published by the National Institutes of Health. The human experiments were implemented under the approval of the Ethics Committee of Cangzhou Central Hospital and conform to the principles of the Declaration of Helsinki. All the participants or their legal guardians signed written informed consent.

### Bioinformatics analysis

A glioma-related gene expression microarray GSE140746 was retrieved from the Gene Expression Omnibus (GEO) database, and the glioma-related gene expression data (TCGA-[glioblastoma multiforme] GBM and TCGA-[lower grade glioma] LGG) were obtained from the TCGA database. Differentially expressed genes in glioma tissue samples were then identified using the Limma package in R language. Venny v.2.1 was utilized to construct a Venn diagram.

### Clinical samples

A total of 25 glioma patients, 15 males and 10 females, aged 27–56 years with a median age of 42 years (14 cases ≤ 42 years, 11 cases >42 years), who underwent glioma resection from 2017 to 2019 in Cangzhou Central Hospital were enrolled. Patients were included in this study when they met the following criteria: 1) with pre-surgical brain MRI and MRS abnormalities indicating glioma and pathologically diagnosed with glioma after surgery; 2) receiving surgical treatment for the first time; 3) with surgically identified accessibility of normal brain tissues from nonfunctional brain areas. Meanwhile, 15 normal brain tissue samples were collected from 15 cases of death caused by brain trauma. All the tissue specimens were frozen in liquid nitrogen and stored at −80°C.

### Cell grouping and transfection

Normal human astrocyte cell line (NHA), four glioma cell lines (SHG-44, A172, T98G, and U251) and the mouse glioma cell line GL261 were purchased from American-type culture collection (ATCC, Manassas, VA). The cells were cultured in serum-free DMEM medium (Lonza, Verviers, Belgium) supplemented with epidermal growth factor (EGF; 20 ng/mL, Peprotech, Rocky Hill, NJ) and basic fibroblast growth factor (FGF; 20 ng/mL, Peprotech) at 37°C and 5% CO_2_.

Cells at the logarithmic phase were trypsinized and seeded into a 6-well plate (1 × 10^5^ cells/well) for 24-h incubation. Upon the cell confluence reaching about 75%, the SHG-44 cells were grouped and transfected according to the protocols of Lipofectamine 2000 (Invitrogen, Carlsbad, CA) with vectors overexpressing MAGT1 with/without U0126 (an ERK inhibitor, DMSO dissolved, working concentration of 5 μM) or harboring short hairpin RNA (shRNA) targeting MAGT1, as listed in Supplementary Table S1. The vectors were commercially synthesized (GenePharma, Shanghai, China) and used at the concentration of 50 ng/µL. Three different primer sequences were designed for sh-MAGT1, and the one with the optimal silencing efficiency, as detected by quantitative reverse-transcription polymerase chain reaction (qRT-PCR; Supplementary Figure S1), was selected for subsequent experiments.

### RNA extraction and quantification

Total RNA was extracted from cells utilizing an RNA extraction kit (DP431, Qiagen, Shanghai, China), followed by the measurement of concentration with an ultraviolet spectrophotometer (ND-1000, Nano Drop Technologies, Wilmington, Delaware). The RNA was reversely transcribed into complementary DNA based on protocols of PrimeScript RT reagent Kit (RR037A, Takara, Shiga, Japan). Then, the qRT-PCR was performed using the SYBR-Green PCR kit (FP205, TIANGEN Biotech, Beijing, China) and ABI PRISM7500RT-PCR system (ABI, Foster City, CA), with GAPDH as the internal control. Each sample was repeated in three wells. Involved primers are listed in Supplementary Table S2.

### Western blot assay

Total protein was extracted using radio-immunoprecipitation assay lysis buffer (Beyotime, Haimen, Jiangsu, China), followed by determination of protein concentration utilizing bicinchoninic acid assay kit. Then, the prepared protein sample was separated by 10% sodium dodecyl sulfate polyacrylamide gel electrophoresis, electro-transferred to polyvinylidene fluoride membrane, and blocked for 2 h with 5% bovine serum albumin. Afterward, separated proteins were incubated overnight at 4°C with diluted rabbit primary antibodies, including anti-MAGT1 (1:500, AB90478, Abcam, Cambridge, UK), anti-ERK-1/2 (1:1000, #4695, Cell Signaling Technology [CST], Danvers, MA), anti-p-ERK (1:2000, #4370, CST, anti-PD-L1 (1:1000, #13,684, CST)), and anti-GAPDH (1:10,000, Ab181602, Abcam). After washing, the proteins were further incubated for 1 h with horseradish peroxidase (HRP)-conjugated secondary antibody (1:100, ab6721, Abcam). Protein bands were then visualized by ECL reagent (Beyotime) and quantified utilizing the Image J 1.42 software (National Institutes of Health, Bethesda, MD) with GAPDH as the internal reference.

### MTT assay

An MTT assay was conducted to evaluate cell viability. Cells were seeded into a 96-well plate (5 × 10^3^ cells/well) and incubated for 24 h with 100 µl complete medium, which was then replaced with serum-free medium. Next, the MTT assay was performed on the basis of protocols of the MTT kit (ab211091, Abcam), followed by measurement of the optical density (OD) at 590 nm.

### Flow cytometry

After 48 hours of transfection, cells were exposed to irradiation (2 Gy), incubated for 48 h, and separated with 0.25% EDTA-free trypsin (YB15050057, Yu Bo Biotech, Shanghai, China). Then, harvested cells were placed in a flow tube for centrifugation for twice, and the supernatant was discarded. Subsequently, cells were treated with Annexin-V-FITC Apoptosis Detection Kit (K201-100, Biovision, Mountain View, CA) and HEPES buffer solution (PB180325, Procell, Wuhan, China) following the instructions. Annexin-V-FITC/PI-stained cells were identified as apoptotic cells and detected under a wavelength of 488 nm using the 525 and 620 nm bandpass filters.

### Colony formation assay

The cells were irradiated with the X-RAD320 irradiation system (AGFA, Wilmington, MA). Then, the irradiated and unirradiated cells were prepared into a single-cell suspension, respectively, and seeded into a six-well plate (200 cells/well) for two-week incubation to form colonies. After staining with 0.01% crystal violet (Sigma), the colonies were subjected to microscopic examination, where a colony containing more than 50 cells was considered a viable cell clone. The rate of colony formation and survival fraction were calculated.

### Establishment of a mouse glioma model

Mouse glioma cells (GL261) transduced with firefly luciferase-mCherry-contained lentiviral vectors were injected into the right caudate nucleus of C57BL/6 mice (aged 6–8 weeks) with a dose of 2 μL (2 × 10^4^ cells/per mouse) using the stereotactic injection method as previously described [19,20]. Before the transduction, lentiviral vectors were diluted with PBS into different titers, and the optimal titer was selected through determination of fluorescence intensity in transduced GL261 cells. For the formal transduction, GL261 cells were seeded in 24-well plates (5 × 10^4^ cells/well), and corresponding lentivirus solution was added upon cells entering the logarithmic phase, along with 10 ug/mL Polybrene (H8761, Solarbio, Beijing, China) added to facilitate the transduction. The medium was renewed 16–24 h later and stably transduced cells were screened 72 h later using 1 µg/mL puromycin (A1113803, Invitrogen), followed by qRT-PCR detection of the transduction efficiency. Notably, the cells were mixed with Matrigel to prevent their migration.

### Immunohistochemistry

After antigen retrieval, the sections of glioma tissues were incubated overnight at 4°C with rabbit primary antibodies including anti-Ki-67 (ab18850, 1:200, Abcam), anti-PD-L1 (#13,684, 1:200, CST), and anti-p-ERK (#4370, 1: 200, CST). After washing, the sections were incubated for 30 min at room temperature with secondary IgG antibody (#ab67211, 1:400, Abcam), followed by visualization using DAB and hematoxylin. Stained sections were then observed with an optical microscope, and images were photographed for analysis. For image analysis, the ‘IHC Profiler’ plugin of the ImageJ software was utilized for deconvolution [21].

### Statistical analysis

Data in this study were processed utilizing SPSS v.21.0 (IBM, Chicago, IL) software. Measurement data were summarized as mean ± standard deviation. Unpaired t-test was applied for comparison between the data of two groups. One-way analysis of variance (ANOVA) with Tukey’s post-hoc test was performed for comparison among data of multiple groups, and repeated measures ANOVA with Tukey’s post hoc test were compared at different time points. The correlation between PD-L1 and MAGT1 was evaluated by Pearson analysis. Moreover, *p* < 0.05 indicated a statistically significant difference.

## Results

### Overexpression of MAGT1 in glioma tissues is associated with poor prognosis in patients with glioma

With the aim to establish novel molecular mechanisms underlying glioma cell radioresistance, this study proposed a hypothesis that MAGT1 may regulate the ERK/MAPK signaling pathway and PD-L1 in glioma. The expression pattern of MAGT1 in glioma tissues was first predicted by microarray profiling and then verified in glioma cells.

**Figure 1.**
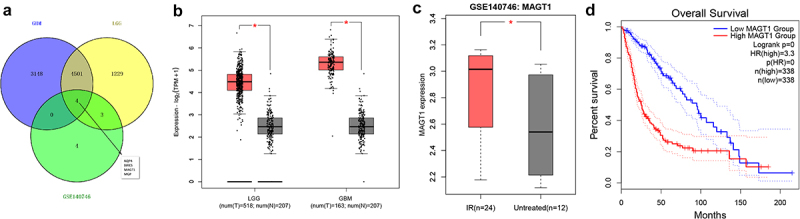
The expression of MAGT1 is upregulated in glioma tissues. A: Differentially expressed genes identified in TCGA-GBM and TCGA-LGG datasets and GSE140746 microarray difference, and a Venn diagram for intersection of upregulated genes. B: MAGT1 level in the TCGA-GBM and TCGA-LGG datasets, with red indicating glioma (GBM/LGG) and gray indicating normal control (* *p* < 0.05). C: The expression of MAGT1 in GSE140746 microarray, with red indicating glioma tissues after radiotherapy, and gray indicating untreated glioma tissues (* *p* < 0.05); D: Kaplan-Meier survival curve for analysis on the correlation between MAGT1 expression and clinical prognosis in patients with glioma.

In bioinformatics analysis, we took the intersection of upregulated genes identified in TCGA-GBM dataset, TCGA-LGG dataset, and the GSE140746 microarray, four glioma-related candidate genes were thereupon obtained, namely AQP4, BIRCS, MAGT1, and MGP (Figure 1)). Analysis on the TCGA-GBM and TCGA-LGG datasets unveiled the overexpression of MAGT1 in glioma tissues (Figure 1)), and that on glioma-related GSE140746 microarray indicated higher expression of MAGT1 in glioma tissue after radiotherapy as compared with untreated glioma tissues (Figure 1)). Kaplan–Meier survival curves further revealed the positive correlation between MAGT1 expression and the poor prognosis of patients with glioma in TCGA-GBM and TCGA-LGG cohorts (Figure 1)). Collectively, our bioinformatics analysis revealed the overexpression of MAGT1 occurring in glioma tissues and correlated it with the poor prognosis in patients with glioma. Thus, we selected MAGT1 as our research focus.

### MAGT1 is highly expressed in glioma cells

**Figure 2.**
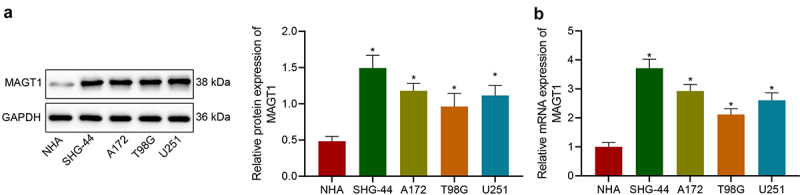
The expression of MAGT1 in four glioma cell lines and a normal human astrocyte cell line (NHA). A: Western blot to determine MAGT1 protein expression in four glioma cell lines (SHG-44, A172, T98G, and U251) and NHA. B: qRT-PCR to measure the level of MAGT1 mRNA in glioma cells and NHA. * *p* < 0.05 versus the NHA cells. Each cell experiment was repeated three times.

We then investigated the expression of MAGT1 in a normal human astrocyte (NHA) cell line and four glioma cell lines (SHG-44, A172, T98G and U251). According to the results, MAGT1 expression was obviously upregulated in the four glioma cell lines, as reflected by both Western blot (Figure 2)) and qRT-PCR (Figure 2)) assays. Herein, our data substantiated the overexpression of MAGT1 in glioma cells.

### MAGT1 promotes cell clonogenic potential and augments the resistance of glioma cells to irradiation

**Figure 3.**
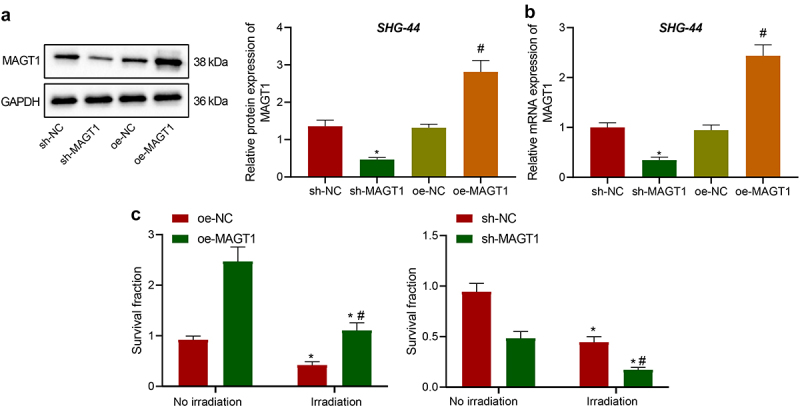
The effects of MAGT1 on clonogenic potential and radioresistance of glioma cells. A: Western blot to determine the protein level of MAGT1 in SHG-44 cells overexpressing/silencing MAGT1 (* *p* < 0.05 versus the sh-NC group. # *p* < 0.05 versus the oe-NC group); B: qRT-PCR detection of MAGT1 mRNA expression level in SHG-44 cells overexpressing/silencing MAGT1 (* *p* < 0.05 versus the sh-NC group. # *p* < 0.05 versus the oe-NC group); C: Colony formation assay to detect the clonogenic potential of irradiated/unirradiated SHG-44 cells overexpressing/silencing MAGT1. (* *p* < 0.05 versus the sh-NC group or oe-NC group without irradiation; # *p* < 0.05 versus the oe-NC group or sh-NC group under irradiation). Each cell experiment was repeated three times.

Following the identification of the upregulated MAGT1 expression in glioma tissues and cells, we then explore the effect of MAGT1 on radioresistance of glioma cells. Among the four glioma cell lines, we selected for subsequent experiments the SHG-44 cell line, which presented with the highest MAGT1 expression (Figure 2). After cell transfection, MAGT1 expression in transfected SHG-44 cells was determined. As a result, the expression of MAGT1 was down-regulated in response to sh-MAGT1 and upregulated in response to oe-MAGT1 treatment (Figure 3).

Further, colony formation ability of these cells was assessed under irradiation (2 Gy) to explore the effects of MAGT1 on glioma cell resistance to irradiation. It was shown that MAGT1 restoration led to the enhanced clonogenic potential of irradiated SHG-44 cells, whereas MAGT1 silencing led to the opposite, indicating that MAGT1 increased the resistance of SHG-44 cells to radiation; consistent results were observed in unirradiated cells, indicating that MAGT1 could augment the clonogenic potential of SHG-44 cells (Figure 3)).

In summary, MAGT1 augmented the clonogenic potential of glioma cells and contributed to the resistance of glioma cells to irradiation.

### MAGT1 stimulates the viability of glioma cells by activating the ERK/MAPK signaling pathway

**Figure 4.**
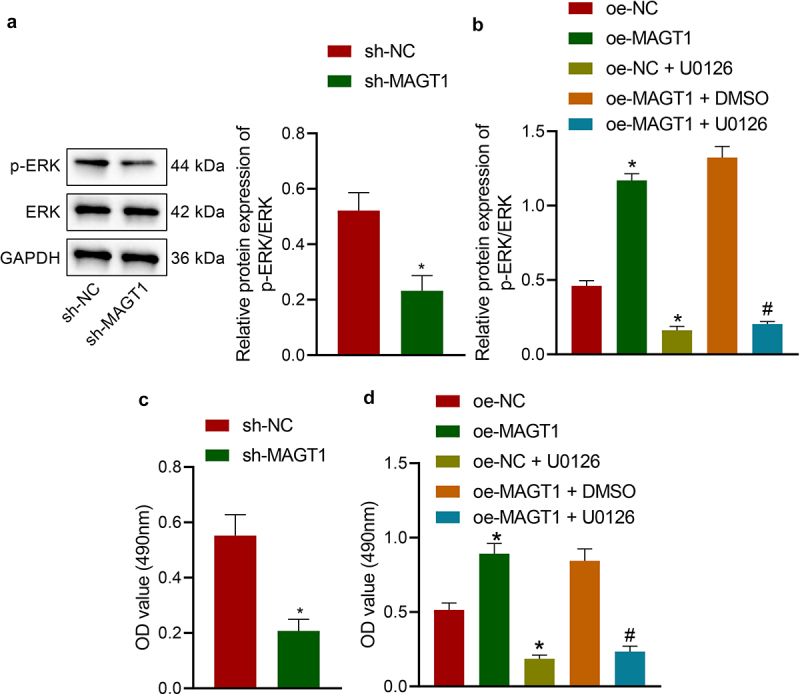
MAGT1 regulates the ERK/MAPK signaling pathway and thus affect the proliferation of glioma cells. A: Western blot to determine the extent of p-ERK and protein levels of ERK in cells silencing MAGT1. B: Western blot to determine the extent of p-ERK and protein levels of ERK in cells overexpressing MAGT1 with/without U0126-induced ERK inhibition; C-D: MTT assay to detect the viability of cells in response to silencing MAGT1 (c), or MAGT1 overexpression alone or in combination with U0126 treatment (d). * *p* < 0.05 versus the sh-NC/oe-NC group, # *p* < 0.05 versus the oe-MAGT1 + DMSO group. Each cell experiment was repeated three times.

Afterward, we managed to validate the hypothesis that MAGT1 may affect the viability and radioresistance of glioma cells by regulating the ERK/MAPK signaling pathway. We first determined the expression of ERK protein and found that glioma cells silencing MAGT1 presented reduced extent of p-ERK but almost unchanged level of total ERK protein (Figure 4)). Next, the extent of p-ERK was revealed to be elevated in glioma cells in the presence of MAGT1 overexpression, and this elevation was abrogated when MAGT1 overexpression was combined with ERK inhibitor U0126 (Figure 4)). Further, MAGT1 silencing resulted in suppressed viability of the cells and MAGT1 overexpression led to the opposite; meanwhile, the glioma cell viability-promoting property of MAGT1 overexpression was reversed by its combination with U0126-induced ERK inhibition (Figure 4). Moreover, the inhibiting effects of U0126 itself on ERK expression and glioma cell viability were confirmed by variations between the oe-NC and the oe-NC + U0126 groups (Figure 4). Taken together, MAGT1 may stimulate the viability of glioma cells *via* activation of the ERK/MAPK signaling pathway.

**MAGT1 increases the resistance of glioma cells to irradiation by activating the ERK/MAPK signaling pathway**

**Figure 5.**
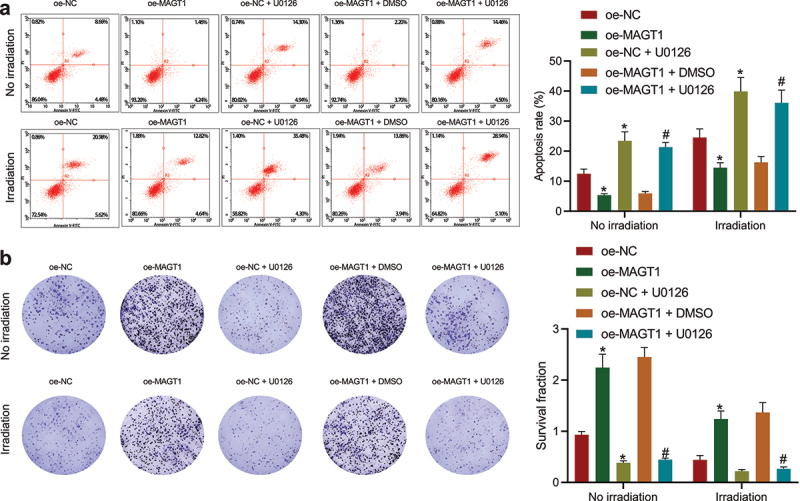
MAGT1 enhances the proliferation and radioresistance of glioma cells through the ERK signaling pathway. A: Flow cytometry to detect the apoptotic rate of SHG-44 cells in response to MAGT1 overexpression alone or its combination with U0126-induced ERK inhibition. B: Colony formation assay to evaluate SHG-44 cell clonogenic potential in response to MAGT1 overexpression alone or its combination with U0126-induced ERK inhibition. * *p* < 0.05 versus the oe-NC group. # *p* < 0.05 versus the oe-MAGT1 + DMSO group. Each cell experiment was repeated 3 times.

Since the aforementioned results have illuminated the promoting effect of MAGT1 on glioma cell proliferation, we then investigated the regulatory role of MAGT1 in radioresistance of glioma cells (SHG-44). As shown by flow cytometry, the apoptosis rate was obviously down-regulated by MAGT1 overexpression in both irradiated and unirradiated glioma cells, which could then be reversed by additional U0126-induced ERK inhibition (Figure 5)). Consistently, the increase in the clonogenic potential caused by MAGT1 overexpression alone was abrogated when it was combined with U0126 treatment, either under or not under irradiation (Figure 5)). Collectively, our data suggested that MAGT1-mediated activation of the ERK/MAPK signaling pathway increased not only the cell proliferation but also the radioresistance in SHG-44 glioma cells.

**Blocking the activation of the ERK/MAPK signaling pathway diminishes the expression of PD-L1 and thus represses glioma cell growth**

**Figure 6.**
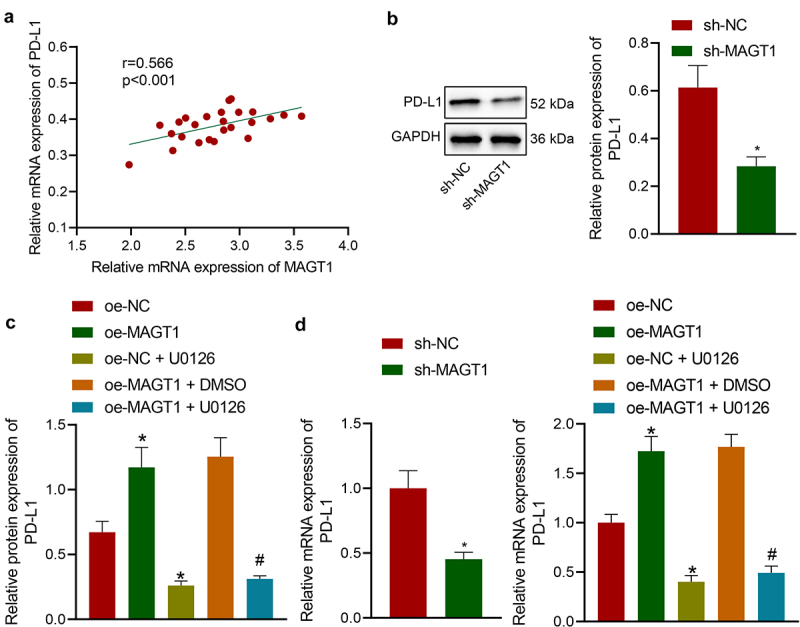
MAGT1 elevates PD-L1 expression by activating ERK. A: The expression of MAGT1 and PD-L1 in clinically collected tumor tissues from glioma patients (n = 50) determined by qRT-PCR, and their correlation assessed with Pearson’s correlation analysis. B: Western blot to determine the protein level of PD-L1 in SHG-44 cells silencing MAGT1. C: Western blot to determine the protein level of PD-L1 in SHG-44 cells in response to oe-MAGT1 and U0126 alone or in combination; D: qRT-PCR to measure the mRNA level of PD-L1 in SHG-44 glioma cells in response to sh-MAGT1 (left panel) or oe-MAGT1 and U0126 alone or in combination (right panel). * *p* < 0.05 versus the sh-NC/oe-NC group. # *p* < 0.05 versus the oe-MAGT1 + DMSO group. Each cell experiment was repeated 3 times.

The aforementioned results have revealed that MAGT1 could upregulate the phosphorylation level of ERK. Herein, we then tried to validate the interaction between MAGT1 and ERK-mediated PD-L1. Through Pearson’s correlation analysis, a positive correlation between MAGT1 expression and PD-L1 expression was identified in clinically collected tumor tissues from glioma patients (Figure 6)). We then determined the PD-L1 protein level in glioma cells with manipulated MAGT1 expression. It was found that SHG-44 cells exhibited down-regulated protein expression of PD-L1 in the presence of MAGT1 silencing or U0126 treatment; MAGT1 overexpressing-cells exhibited upregulated level of PD-L1, and U0126-induced ERK inhibitor reversed the oe-MAGT1-induced upregulation (Figure 6). The results of qRT-PCR were consistent with those of Western blot assay (Figure 6)). Taken together, MAGT1 enhanced PD-L1 expression by activating the ERK/MAPK pathway, thereby facilitating glioma cell growth.

### *MAGT1 accelerates the tumor formation of glioma* in vivo

**Figure 7.**
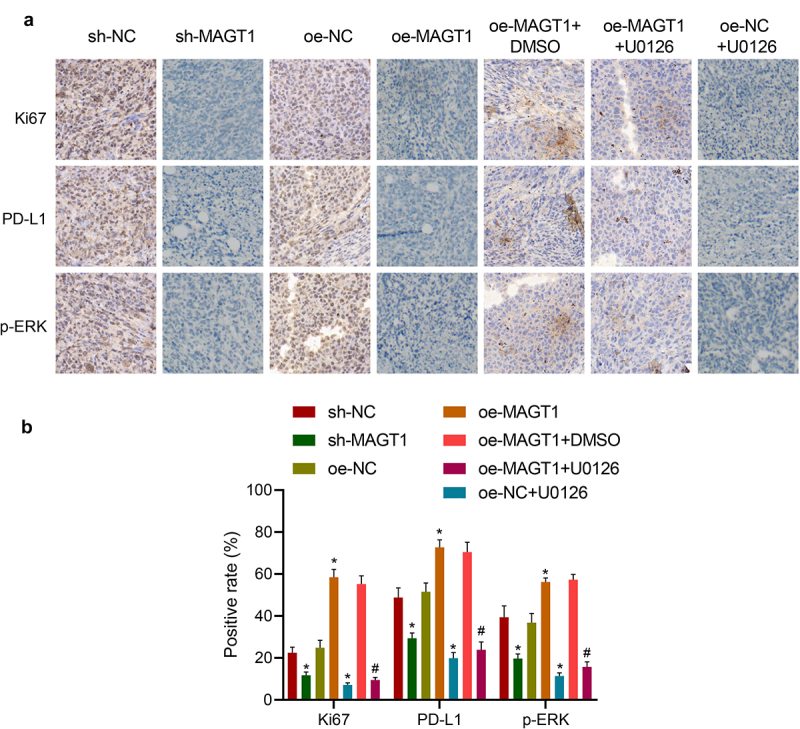
MAGT1 accelerates the tumor formation of glioma cells in mice. A-B: Representative images (a) of immunohistochemical staining to detect expression of Ki-67, PD-L1 and extent of p-ERK in glioma tissues and corresponding quantification (b) using the ‘IHC Profiler’ plugin of the ImageJ software. n = 6 for each group. * *p* < 0.05 versus the sh-NC or oe-NC group, # *p* < 0.05 versus the oe-MAGT1 + DMSO group.

Following the *in vitro* experiments, we then moved to investigate the effect of MAGT1 on *in vivo* glioma formation in a mouse model established by stereotactic injection GL261 cells. According to immunohistochemical staining, the positive rate of Ki-67, a well-recognized proliferation-related factor, was down-regulated in response to either MAGT1 knockdown or U0126 treatment, accompanied by repressed extent of p-ERK and expression of PD-L1. Besides, restoration of MAGT1 led to elevated levels of Ki-67 and PD-L1 and extent of p-ERK, which were negated by additional treatment with U0126 (Figure 7). Altogether, our *in vivo* data, consistent with *in vitro* results, substantiated that MAGT1 could upregulate the expression of PD-L1 by activating the ERK signaling pathway, thereby stimulating the growth of glioma cells *in vivo*.

## Discussion

Glioma remains one of the most treatment-resistant cancers with alarmingly poor clinical outcomes [2]. In the present study, we elucidated that MAGT1 in glioma could activate the ERK/MAPK signaling pathway and upregulate PD-L1 expression, thereby contributing to the growth and radioresistance of glioma cells.

Our initial findings of the bioinformatics analysis identified MAGT1 as a candidate gene related to glioma due to its overexpression in glioma tissues, which was correlated with the poor prognosis of patients with glioma. We then validated that MAGT1 was also overexpressed in glioma cells. Our findings corroborate previous studies where microRNA-199a-5p was revealed to repress glioma progression through impeding MAGT1 expression [6] and the circ_0002755/miR-628-5p/MAGT1 axis was found to participate in the progression of glioma [22]. Furthermore, our data substantiated that MAGT1 may trigger the proliferation of glioma cells and contribute to the resistance of glioma cells to irradiation. In relation to this, it has been established that MagT1 overexpression in DT40 cells could stimulate cell growth [23]. Additionally, since MagT1 is involved in protein glycosylation as a subunit of the oligosac-charyltransferase complex, it is speculated that silencing MagT1 affects post-translational modification of proteins participating in cell proliferation [24,25]. Besides, the upregulation expression of MAGT1 has been correlated with the aggressiveness as well as poor prognosis of colorectal cancer [26].

Further to explore the downstream mechanisms, we unraveled that MAGT1 stimulated not only the cell proliferation but also the radioresistance in glioma cells by activating the ERK/MAPK signaling pathway. A previous study also indicated that MAGT1 could activate the ERK signaling pathway [7]. In agreement with our findings, there is a growing body of evidence supporting the regulatory role of the ERK/MAPK signaling pathway in glioma. For instance, MiR-130b was highlighted as an oncogene in glioma *via* regulating the ERK/MAPK signaling pathway [27]; interference of angiopoietin-like protein 2 was found to repress glioma cell proliferation and invasion through blocking the ERK/MAPK signaling pathway [8]; and Cedrelone exhibited antitumor activity in temozolomide-resistant human glioma partially based on modulation of the ERK/MAPK signaling pathway [28]. Concerning radioresistance, it has been established that ERK5 could increase radioresistance of lung cancer cells through stimulating the DNA damage response [29], and activation of ERK has been revealed as a rescue mechanism after irradiation in head and neck squamous cell carcinoma [30].

Following the MAGT1/ERK/MAPK regulatory axis, our data then illuminated MAGT1 triggered the expression of PD-L1 by activating the ERK/MAPK pathway. Our finding partly corroborates a previous report where ERK/MAPK signaling pathway has been highlighted as a mediator of PD-L1 expression in mesothelioma [31]. Notably, the PD-1/PD-L1 immunoregulatory axis has been reported to confer a promoting effect on invasion of glioblastoma multiforme cells in the brain tissues [17]. Blocking the PD-1/PD-L1 pathway is emerging as a potential therapeutic regimen for glioma [32]. Ene *et al*. suggested anti-PD-L1 antibody to be a contributor to radiation-induced abscopal response *via* direct macrophage activation in glioblastoma [33]. Suppression of PD-L1 and its pro-tumoral activities has been unveiled to modulate the self-renewal and growth capacities of glioma cells, thus conferring a role in glioma resistance [34]. In line with the prior documentation, our *in vivo* experiments substantiated that MAGT1 could enhance the expression of PD-L1 by activating the ERK signaling pathway, thereby contributing to the growth and radio-resistance of glioma cells.

## Conclusion

**Figure 8.**
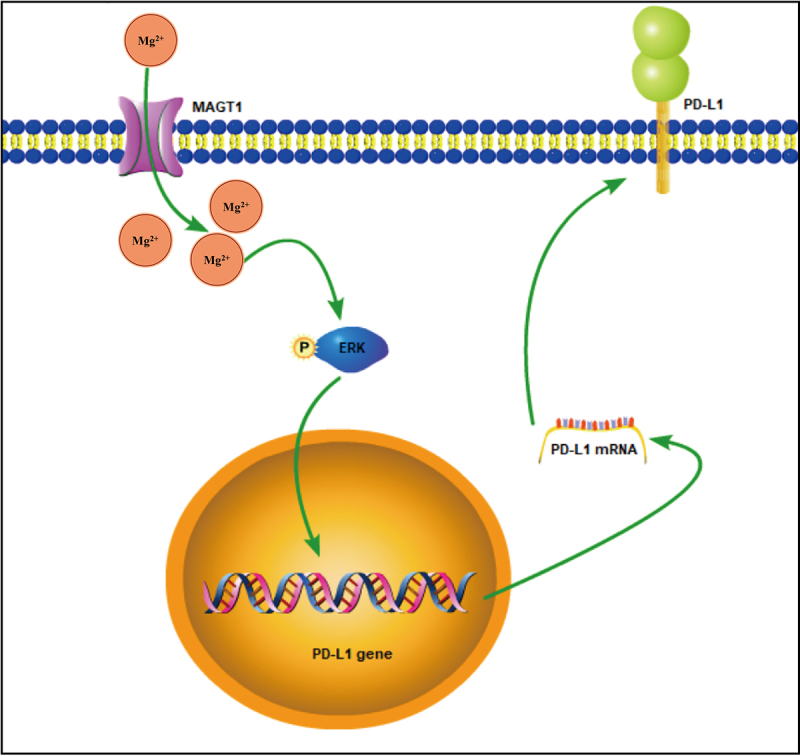
The mechanism graph of the regulatory network and function of MAGT1 in glioma. MAGT1 promotes the growth and radioresistance of glioma cells through ERK/MAPK signaling pathway-mediated upregulation of PD-L1 expression.

Taken together, the data acquired in this study led to the conclusion that MAGT1 overexpression contributed to the growth and radioresistance of glioma cells through ERK/MAPK signaling pathway-mediated upregulation of PD-L1 expression (Figure 8). Our findings deepen our standing of the tumorigenesis of glioma and provide a theoretical basis for the development of novel targeted therapies for glioma. Also, by indicating the involvement of the MAGT1/ERK/MAPK axis in radioresistance of glioma cells, this study provides potential adjuvant therapies for enhancing the efficacy of radiotherapy in clinical treatment for glioma. However, this study was limited by the insufficiency of *in vivo* results, which should be supplemented in future studies to further substantiate *in vitro* findings.

## Supplementary Material

Supplemental Material

## Data Availability

The datasets generated/analyzed during the current study are available.
