# Supplementary material for: Oncogenic magnesium transporter 1 upregulates programmed death-1-ligand 1 expression and contributes to growth and radioresistance of glioma cells through the ERK/MAPK signaling pathway
Source: Bioengineered. 2022 Apr 13;13(4):9575–87. doi: 10.1080/21655979.2022.2037214 (PMC9161830; doi:10.1080/21655979.2022.2037214)
Supplement: Supplemental Material [file KBIE_A_2037214_SM0126.zip › supplementary/Supplementary Tables.docx]

**Supplementary Table 1 Cell grouping and transfection**

| Groups | Transfected plasmids |
| --- | --- |
| sh-NC | Plasmids harboring NC shRNA |
| sh-MAGT1 | Plasmids harboring shRNA targeting MAGT1 |
| oe-NC | NC overexpression plasmids |
| oe-MAGT1 | MAGT1 overexpression plasmids |
| oe-MAGT1+DMSO | MAGT1 overexpression plasmids dissolved with DMSO |
| oe-MAGT1+U0126 | MAGT1 overexpression plasmids with U0126 |

**Note:** MAGT1, magnesium transporter 1; NC, negative control; shRNA, short hairpin RNA

**Supplementary Table 2** **Primer sequences for qRT-PCR**

| Genes | Primer sequences |
| --- | --- |
| MAGT1 | Forward: 5’-GGGCTTTTGCAGCTTTGTGT-3’ |
|  | Reverse: 5’-AAACTGGGCTTGACTGCTTC-3’ |
| PD-L1 | Forward: 5’-CAATGTGACCAGCACACTGAGAA-3’ |
|  | Reverse: 5’-GGCATAATAAGATGGCTCCCAGAA-3’ |
| ERK-1 | Forward: 5’-CCTGCGACCTTAAGATTTGTGATT-3’ |
|  | Reverse: 5’-CAGGGAAGATGGGCCGGTTAGAGA-3’ |
| ERK-2 | Forward: 5’-GCGCGGGCCCGGAGATGGTC-3’ |
|  | Reverse: 5’-TGAAGCGCAGTAAGATTTTT-3’ |
| GAPDH | Forward: 5’-CATGGCACCGTCAAGGCTGA-3’ |
|  | Reverse: 5’-ACGCCAGTGGACTCCACGACGT-3’ |

**Note:** MAGT1, magnesium transporter 1; ERK, extracellular signal-regulated kinase; GAPDH, glyceraldehyde-3-phosphate dehydrogenase; PD-L1, programmed death-1-ligand 1
