# Supplementary figures and images for: Oncogenic magnesium transporter 1 upregulates programmed death-1-ligand 1 expression and contributes to growth and radioresistance of glioma cells through the ERK/MAPK signaling pathway
Source: Bioengineered. 2022 Apr 13;13(4):9575–87. doi: 10.1080/21655979.2022.2037214 (PMC9161830; doi:10.1080/21655979.2022.2037214)

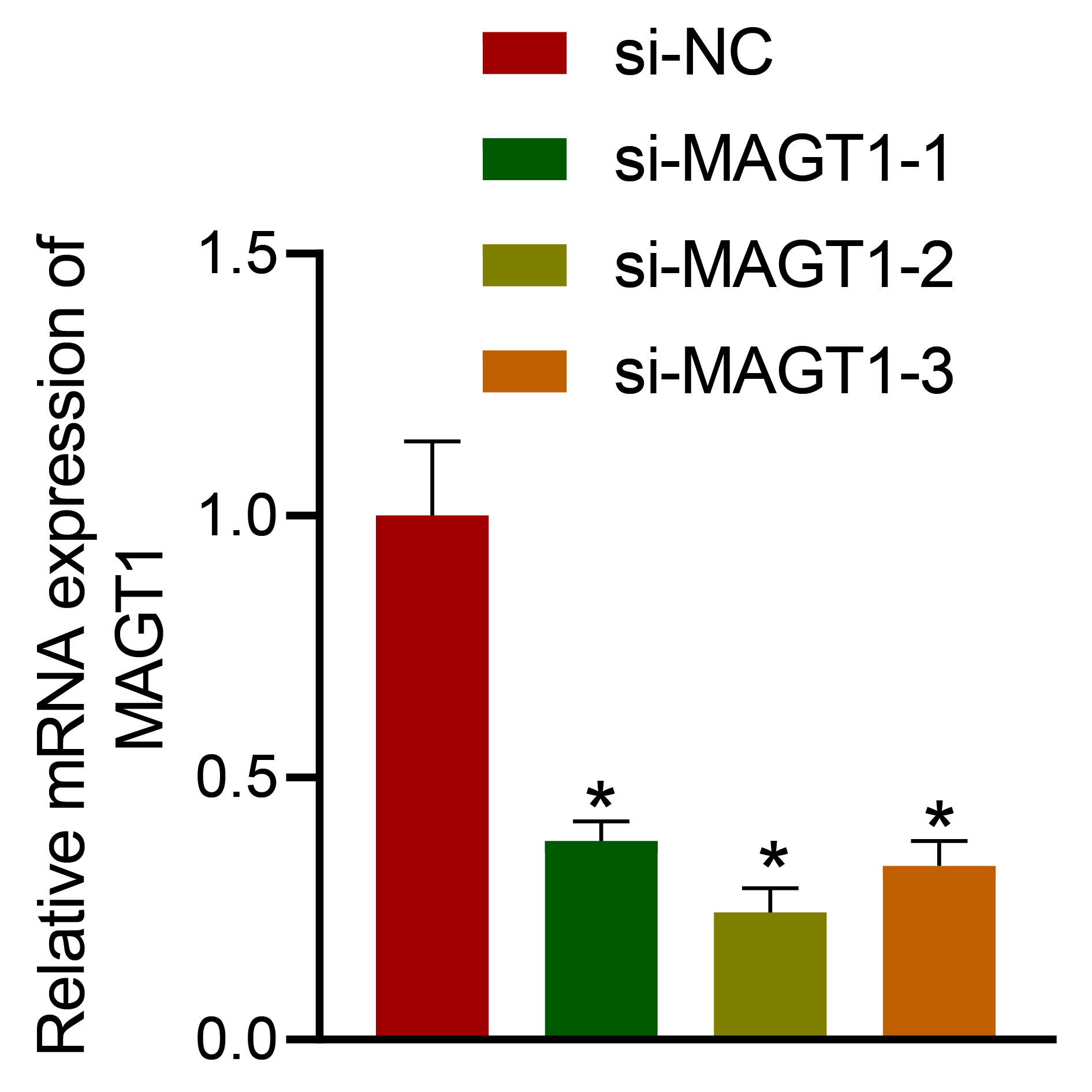

Supplement: Supplemental Material [file KBIE_A_2037214_SM0126.zip › supplementary/Figure S1.jpg]
